# Obstetric Aphorisms

**Published:** 1873-02

**Authors:** 


*Obstetric Aphorisms: For the Use of Students commencing Midwifery Practice.* By Joseph Griffiths Swayne, M. D. Second American from the fifth English edition with additions. By E. R. Hutchins, M. D. Philadelphia: Henry C. Lea, 1873. Buffalo: T. Butler & Son.

In this little work, of some one hundred and eighty pages, we have given in an abridged and concise form the principal points of the obstetric art, and to the busy practitioner who is for the time forgetful of the exact terms and technicalities of obstetric language, it will serve as a convenient and compact book of reference. We are not to be understood to advocate the use of handbooks, &c., to assist the professional man in his diagnosis and treatment of disease, but often in the hurry of busy practice where the practitioner wishes to refresh his mind on some simple and unimportant point, reference to some book of this kind will serve his purpose equally well with the larger and more thorough treatises. In no department more than in obstetrics is the young practitioner fearful of mistakes, and to one well informed in the principles of his art we know of no book which will better serve the purpose of an occasional remembrancer than the one now before us.

